# Tumor necrosis factor in lung cancer: Complex roles in biology and resistance to treatment^1^^2^

**DOI:** 10.1016/j.neo.2020.12.006

**Published:** 2020-12-26

**Authors:** Ke Gong, Gao Guo, Nicole Beckley, Yue Zhang, Xiaoyao Yang, Mishu Sharma, Amyn A. Habib

**Affiliations:** a Department of Neurology, University of Texas Southwestern Medical Center, Dallas, TX, USA; b Harold C. Simmons Comprehensive Cancer Center, University of Texas Southwestern Medical Center, Dallas, TX, USA; c VA North Texas Health Care System, Dallas, TX, USA

**Keywords:** TNF, NSCLC, EGFR inhibition, Therapeutic resistance, Immunotherapy, TCGA, BATTLE, Biomarker-integrated Approaches of Targeted Therapy for Lung Cancer Elimination, EGFRwt, EGFR wild type, GBM, glioblastoma, HR, hazard ratio, LPS, lipopolysaccharides, NSCLC, non-small cell lung cancer, OS, overall survival, PCA, principal component analysis, TCGA, The Cancer Genome Atlas, TKIs, tyrosine kinase inhibitors, TMZ, temozolomide, TNF, tumor necrosis factor, TNFR1, TNF receptor 1, TNFR2, TNF receptor 2, TNFSFs, TNF superfamily ligand

## Abstract

Tumor necrosis factor (TNF) and its receptors are widely expressed in non-small cell lung cancer (NSCLC). TNF has an established role in inflammation and also plays a key role in inflammation-induced cancer. TNF can induce cell death in cancer cells and has been used as a treatment in certain types of cancer. However, TNF is likely to play an oncogenic role in multiple types of cancer, including NSCLC. TNF is a key activator of the transcription factor NF-κB. NF-κB, in turn, is a key effector of TNF in inflammation-induced cancer. Data from The Cancer Genome Atlas database suggest that TNF could be a biomarker in NSCLC and indicate a complex role for TNF and its receptors in NSCLC. Recent studies have reported that TNF is rapidly upregulated in NSCLC in response to targeted treatment with epidermal growth factor receptor (EGFR) inhibition, and this upregulation leads to NF-κB activation. The TNF upregulation and consequent NF-κB activation play a key role in mediating both primary and secondary resistance to EGFR inhibition in NSCLC, and a combined inhibition of EGFR and TNF can overcome therapeutic resistance in experimental models. TNF may mediate the toxic side effects of immunotherapy and may also modulate resistance to immune checkpoint inhibitors. Drugs inhibiting TNF are widely used for the treatment of various inflammatory and rheumatologic diseases and could be quite useful in combination with targeted therapy of NSCLC and other cancers.

## Introduction

Tumor necrosis factor (TNF) is a cytokine that plays a central role in inflammation, the immune response, and in cancer [1], [2], [3], [4], [5]. TNF is a key regulator of inflammation in rheumatoid arthritis and other inflammatory diseases, and TNF antagonists are commonly used to treat inflammatory conditions [6], [7]. In relation to cancer biology, TNF was initially reported as a serum factor that induced necrosis of tumors [8]. However, the therapeutic use of TNF has been severely limited by toxicity and endotoxic shock. Furthermore, a number of studies have identified an oncogenic role for TNF in inflammation related cancer [9], [10], [11]. Thus, depending on the cellular context, TNF can promote cell death or tumor growth [12], [13], [14]. More recent studies have also identified a potential role for TNF in promoting resistance to both targeted treatment as well as immunotherapy [15], [16], [17], [18], [19], [20], [21], [22]. TNF is produced by a variety of tissues and is inducibly expressed in response to inflammatory stimuli such as lipopolysaccharides (LPS). TNF binds to its cognate receptors, TNF receptor 1 (TNFR1) or TNF receptor 2 (TNFR2), and activates a number of inflammatory signaling networks. TNFR1 is expressed widely, while TNFR2 expression is mostly limited to immune cells and endothelial cells [23], [24]. Both the cytokine and its receptors TNFR1 and TNFR2 are reported to be broadly expressed in lung cancer [25], [26]. Importantly TNF is known to be secreted by malignant cells [9] as well as cells in the tumor microenvironment, and there is experimental evidence from a variety of models that TNF can promote the growth of tumors [27], [28]. In this review, we discuss evidence for an oncogenic role of TNF in cancer with a focus on non-small cell lung cancer (NSCLC). We also discuss the role of TNF in promoting resistance to targeted treatment and immunotherapy with checkpoint inhibitors.

## TNF as a biomarker in lung cancer in the Cancer Genome Atlas and Biomarker-integrated Approaches of Targeted Therapy for Lung Cancer Elimination databases

**Figure 1:**
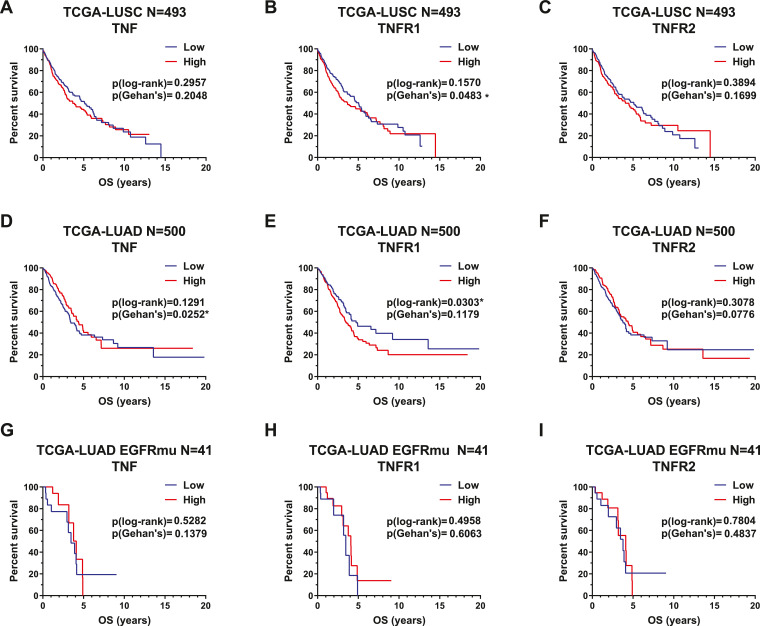
TNF affects TCGA-NSCLC patient survival. (A−C) The Cancer Genome Atlas Lung Squamous Cell Carcinoma (TCGA-LUSC) patients with RNAseq and survival data were divided into high-50% and low-50% groups by TNF, TNFR1, and TNFR2 mRNA levels and the effect on overall survival was examined. Kaplan–Meier (KM) survival curves were drawn and compared by log-rank test and Gehan's test. (D−F) 500 primary TCGA-Lung Adenocarcinoma (TCGA-LUAD) patients’ RNAseq and survival data was analyzed. (G−I). Among the 500 primary TCGA-LUAD patients, there are 41 patients harboring EGFR-activating mutation (with L858R or exon 19 deletion, but without T790M mutation). Roles of TNF/TNFR1/TNFR2 on these patients’ survival data was examined. *P* <0.05 of log-rank was considered statistically significant. Analysis was performed by GraphPad 9. TCGA, The Cancer Genome Atlas; TNF, tumor necrosis factor; TNFR1, TNF receptor 1; TNFR2, TNF receptor 2.

An examination of the the Cancer Genome Atlas (TCGA) database reveals that for the squamous cell subset of NSCLC lung squamous cell carcinoma (LUSC), TNF shows a trend as an oncogene, as patients with higher TNF mRNA levels are associated with shorter overall survival (OS), although this correlation does not reach statistical significance (Figure 1A). Moreover, TNFR1 is significantly oncogenic with a *P* value <0.05 in Gehan's test (Figure 1B), and TNFR2 trends to promote tumor growth with *P* >0.05. (Figure 1C). Gehan's test places more weight on the earlier death events than the log-rank test, whereas the log-rank test equally judges all time points [29]. Therefore, this may indicate that TNF/TNFRs may affect OS in LUSC early after diagnosis, which is similar to progression-free survival (PFS). In conclusion, the TCGA-LUSC data suggests that TNF/TNFR1/TNFR2 expression in tumors have weak adverse prognostic effects on OS. The mechanism could be due to TNF/TNFR1/TNFR2 signals promoting tumor growth, evading immune attacks, or triggering adverse effects in patients.

The adenocarcinoma subset of NSCLC (lung adenocarcinoma (LUAD)) includes patients with EGFR activating mutations, the prevalence of which ranges from 15% in Western populations to up to 50% in Asian populations [30]. Patients with EGFR activating mutations receive treatment with EGFR tyrosine kinase inhibitors (TKIs) since this treatment is effective in early disease control in these patients [31], [32]. Overall, from the TCGA-LUAD database, survival analysis reflects conflicting trends, with Gehan's assay demonstrating a tumor suppressive phenotype of TNF (*P* = 0.025, Figure 1D) and TNFR2 (*P* = 0.07, Figure 1F), while log-rank's test shows a protumorigenic phenotype for TNFR1 (*P* = 0.03, Figure 1E). The conflicting results suggest that the impact of TNF signaling in lung adenocarcinoma is complex. The conflicting outcomes of TNF/TNFRs may be due to the EGFR mutation status, which determines whether EGFR TKIs were used on these patients.

Among the 500 TCGA-LUAD patients with RNAseq and OS data, there are 41 (8%) harboring EGFR activating mutation [18]. For these patients, the roles of TNF/TNFRs are not clear. (Figure 1G–I). A limitation of the TCGA-LUAD database is that it does not provide the drug treatment history, making it difficult to ascertain treatment with EGFR TKIs, which would affect the EGFR mutant patient's survival. Additionally, biomarker profiles may differ between early-stage and advanced lung tumors, and there are limited advanced lung cancer cases in TCGA. Finally, there is a lack of PFS data in TCGA-LUSC and TCGA-LUAD, and the OS data does contain a lot of censored points. In some cancers PFS and OS are strictly related, but in others they are not, and may even be opposite [33]. PFS can represent the direct control of cancer growth, especially under treatment. However, an improvement in PFS does not always bring in improvement on OS, as OS is affected by control of disease and side-effects of treatment. A treatment can control tumor growth and induce severe side-effects, just like a double-edged sword.

Data from the Biomarker-integrated Approaches of Targeted Therapy for Lung Cancer Elimination (BATTLE) trial helps with some of these issues. The BATTLE trial included about 30 advanced-NSCLC (mostly LUAD) patients harboring active EGFR mutation who were treated with erlotinib after the first diagnosis [34]. Both PFS and OS were tracked. PFS has no censored data. An RNA-array assay was performed on the original diagnostic tissue. As we have previously shown, the BATTLE trial data shows TNF and other target genes are tumor promoting in EGFR mutant patients treated with EGFR TKIs, and we confirmed tumor's TNF was elevated by EGFR TKI therapy in vitro, in vivo, and in patients [17].

**Figure 2:**
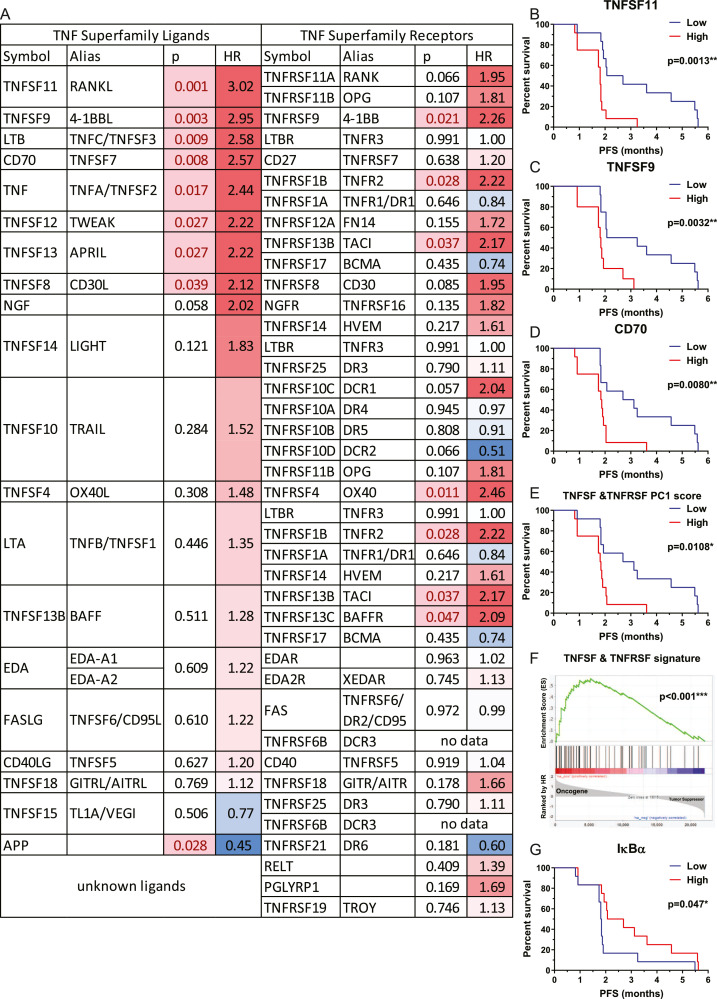
Higher TNF superfamily ligands and receptors determine worse prognosis of NSCLC patients treated with EGFR inhibition. (A) A summary table of survival analysis on TNF superfamily ligands and receptors in the BATTLE trial data (ClinicalTrials.gov NCT00410059 for erlotinib). The higher HR (Hazard Ratio) >1 means the gene would likely promote tumor growth and shorten the progression-free survival (PFS). BATTLE data were provided by the BATTLE group at MD Anderson Cancer Center [17]. (B−D) KM survival curves were drawn on 3 TNF superfamily ligands (TNFSFs) with top 5 HR, the other 2 ligands TNF and LTB was reported in our previous study [17]. (E) The first principle component (PC1) score of current known TNFSFs and TNF superfamily receptors (TNFRSFs) as listed in the Figure 2A for each patient in NCT00410059 was calculated. Patients with higher or lower PC1 scores were compared by KM survival analysis. (F) All gene survival analysis was performed on NCT00410059. All genes were ranked by HR to distinguish oncogenes and tumor suppressors. The enrichment plot by Gene Set Enrichment Analysis (GSEA) shows TNFSF & TNFRSF signature (genes in the Figure 2A) was significantly enriched in oncogenes, genes with higher HR that bring in shorter PFS under erlotinib treatment. (G) KM survival curves of IκBα. *P* <0.05 of log-rank was considered statistically significant. Analysis was performed by GraphPad 9, and GSEA: www.gsea-msigdb.org. BATTLE, Biomarker-integrated Approaches of Targeted Therapy for Lung Cancer Elimination; NSCLC, non-small cell lung cancer; TNF, tumor necrosis factor; TNFSFs, TNF superfamily ligands.

Per the BATTLE trial data, most TNF superfamily genes and NF-κB target genes are oncogenic in EGFR mutant patients treated with EGFR TKIs. Figure 2A lists 20 currently known TNF superfamily ligands (TNFSFs) and their paired receptors (TNFRSFs) [12], [35], and 40% (*n* = 8) of ligands correlate with significantly shortened PFS with *P* <0.05, 50% (*n* = 10), having hazard ratio (HR) >1, consistent with oncogenic roles. Only 2 (10%) may prolong PFS with HR <1, suggesting tumor suppressive roles. Moreover, the majority of TNFRSFs also have HR >1 and paired with these 20 TNFSFs. Three TNFSFs with the top HR and strongest correlation to negative prognosis, TNFSF11/RANKL, TNFSF9/4-1BBL, and TNFSF7/CD70 are shown in Figure 2B–D. Results of the other 2 ligands with the top HRs, TNF and LTB have been published [17]. Overall, by using principal component analysis (PCA) on survival prediction [36], [37], we found the first principle component score of TNFSF & TNFRSF signature (genes listed at Figure 2A) can be used as a biomarker to predict the prognosis of these patients (Figure 2E), *P* = 0.0108. By Gene Set Enrichment Analysis [38], we found the TNFSF & TNFRSF signature was significantly (*P* < 0.001) enriched in oncogenes with higher HR associated with shorter PFS under erlotinib treatment (Figure 2F).

This BATTLE trial data suggests that besides TNF and NF-κB target genes, combination inhibition with the other TNF superfamily members could also be considered with EGFR inhibition treatment, and this may be a useful area for future studies.

## The relationship between TNF and NF-κB

**Figure 3:**
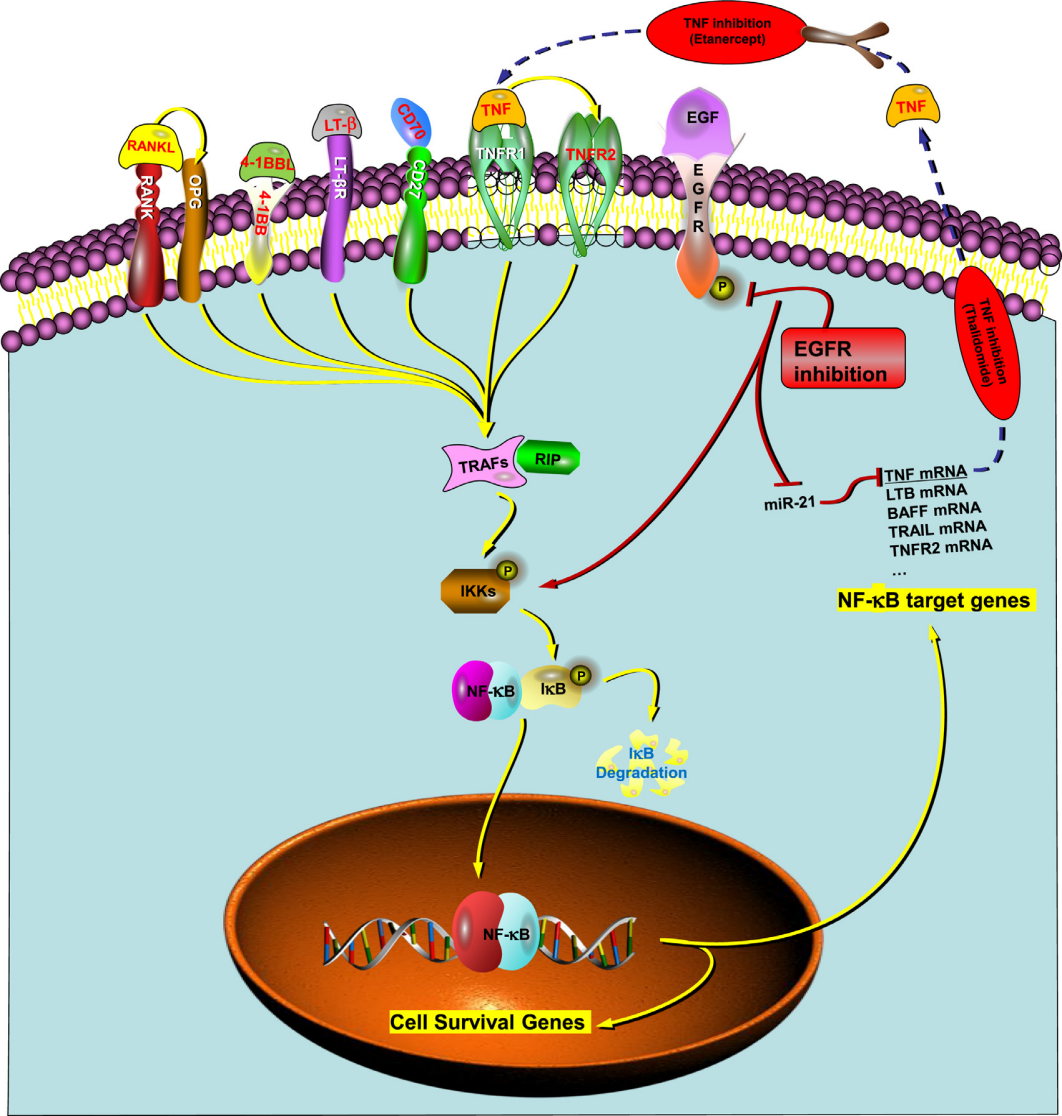
Schematic diagram of TNF signaling and the effect of EGFR and TNF inhibition in NSCLC. Representative TNF superfamily ligands and receptors predicting worse prognosis in the BATTLE trial with statistically significance were labelled in red. BATTLE, Biomarker-integrated Approaches of Targeted Therapy for Lung Cancer Elimination; NSCLC, non-small cell lung cancer; TNF, tumor necrosis factor. (Color version of figure is available online.)

The transcription factor NF-κB plays a central role in inflammation and inflammation-mediated cancer [39], [40]. TNF induced NF-κB activation is triggered by inhibitor of nuclear factor-κB (IκB) kinase (IKK) activation [41], [42], [43]. After TNF binds to TNFR1, IKK is recruited to an activated TNFR1 complex. This complex contains TRADD, RIP, TRAF2/5, and CIAP1/2. Then IKK is activated by RIP1, requiring TAK1 and TAB2/3. On the other hand, when NF-κB is not activated, the cytosolic NF-κB binds with IκB, which has been identified as an inhibitor of NF-κB. The activated IKK phosphorylates IκB and phosphorylated IκB is rapidly degraded by its polyubiquitination. The IκB released NF-κB translocates into the nucleus and promotes transcription of a large number of NF-κB target genes [44] (Figure 3). According to the BATTLE trial data for the erlotinib treatment group, IκBa is a tumor suppressor (Figure 2G). TNF is among the NF-κB target genes [45]. The nuclear-located activated NF-κB binds to the promoter of TNF and increases TNF expression. Most of these NF-kB target genes, including FLIP, BCL-XL, CIAP, XIAP, and others, have anti-apoptosis and pro-survival function. NF-κB is rapidly activated in response to EGFR inhibition in lung cancer and plays an important role in resistance to EGFR targeted treatment [46], [47]. NF-κB activation in response to EGFR inhibition results from increased TNF secretion, leads to NF-κB activation, and increased transcription of TNF in a feed-forward loop [17].

## TNF mediates resistance to targeted treatment in lung cancer

Recent work suggests that TNF may play an important role in resistance to targeted treatment in cancer. The initial results were reported in glioblastoma (GBM). GBM is a devastating cancer with a dismal prognosis. Although there is substantial evidence that aberrant EGFR signaling is widespread in GBM [48], [49], [50], EGFR inhibition, has thus far, failed in GBM. An important mechanism of resistance to EGFR inhibition may be mediated by TNF. EGFR inhibition in experimental models of GBM results in a rapid increase in TNF. TNF activates a JNK-Axl-ERK signaling pathway that mediates resistance to EGFR inhibition [15], [51]. A combined inhibition of EGFR and TNF overcomes the primary resistance to EGFR inhibition in multiple models of experimental GBM [15]. The initial report of a key role for TNF in mediating resistance to EGFR inhibition in GBM has been confirmed by 2 independent studies [19], [20]. Additionally, a follow up study has compared the results of EGFR plus TNF inhibition to the standard of care, temozolomide (TMZ) and demonstrated that EGFR plus TNF inhibition is more effective than TMZ in large subsets of GBM including recurrent GBMs and TMZ resistant GBMs in mouse models suggesting that a combined EGFR plus TNF inhibition may be a viable therapeutic approach in GBM.

TNF may also have an important role in mediating resistance to EGFR inhibition in NSCLC. About 10% to 15% of NSCLC patients in Western populations harbor EGFR activating mutations that render the tumors sensitive to EGFR inhibition, and EGFR TKIs are used as a treatment in this subset of patients. The remaining NSCLC tumors express EGFR wild type (EGFRwt) and do not respond to TNF inhibition exhibiting a primary resistance to EGFR inhibition. TNF upregulation was reported to be a universal and rapid early adaptive response to EGFR inhibition in experimental models of NSCLC regardless of EGFR status [17]. EGFR signaling actively suppresses TNF mRNA levels by inducing expression of miR-21, resulting in decreased TNF mRNA stability (Figure 3). Conversely, EGFR inhibition results in loss of miR-21 and increased TNF mRNA stability [17]. In addition, TNF-induced NF-κB activation leads to increased TNF transcription in a feed-forward loop. A combined inhibition of EGFR plus TNF overcomes the primary resistance of EGFRwt expressing NSCLC cell lines to EGFR inhibition in cell culture and in xenograft studies with cell lines as well as patient derived xenografts [17]. Similar results were obtained in EGFR mutant NSCLC lines that are EGFR TKI sensitive. A combined EGFR plus TNF inhibition was synergistic in these cell lines and xenograft models. NF-κB and lymphotoxin-β were identified as the major effectors of TNF signaling in mediating resistance to EGFR inhibition [17]. Importantly, it was demonstrated that a combined EGFR plus TNF inhibition was also synergistic in an immunocompetent transgenic NSCLC model driven by a doxycycline mediated induction of the L858R EGFR mutation. Other studies have also identified a key role for NF-κB activation in mediating resistance to EGFR inhibition in NSCLC [46], [47], [52], [53]. There may also be a role for PTGS2/COX-2 which is induced by TNF in human lung cancer lines [54], [55], and involved in EGFR inhibition resistance on lung cancer and colorectal cancer [56], [57], [58]. A COX-2 inhibitor, Celecoxib, was used in some clinical trials in combination with EGFR inhibition for treating NSCLC [59], [60]. mPGES-1 has also been identified as a target to overcome acquired resistance to gefitinib in NSCLC cell lines [61].

TNF may also mediate resistance to TKIs in renal cell carcinoma [62]. NF-κB is also involved in another facet of the adaptive response to EGFR inhibition. It was recently shown that EGFR inhibition in NSCLC triggers a biologically significant induction of Type I interferons in NSCLC. Thus, a combined inhibition of EGFR plus Type I interferons is synergistic in both primary and secondary models of resistance to EGFR inhibition in NSCLC [18]. In EGFRwt NSCLC the EGFR inhibition mediated induction of Type I interferons is triggered through an NF-κB dependent pathway [18], [63].

## TNF in immunotherapy for lung cancer

Immunotherapy with checkpoint inhibitors has been an exciting advancement for multiple cancer types including NSCLC. About 20% of patients with NSCLC respond to immunotherapy [64], [65]. However, NSCLCs with EGFR activating mutations appear to be resistant to immunotherapy [66], [67], [68]. Immunotherapy with PD1 or CTLA4 antibodies or a combination of the 2 is associated with a significant toxicity that is associated with inflammation and immune–related adverse events that may involve TNF production and is commonly treated with glucocorticoids and also with anti-TNF therapies [69], [70], [71]. While this appears to be an effective treatment, experimental studies have suggested that inhibition of TNF may help to overcome resistance to immune checkpoint treatments in murine experimental models [21], [22]. The outcome of anti-TNF treatment appears to be more complex in patients [72]. While some reports in small numbers of patients have suggested that anti-TNF treatment may be beneficial in combination with immune checkpoint therapy [73], [74], other studies have indicated that administration of anti-TNF therapy does not affect the outcome of immune checkpoint treatment [75], [76]. A recent comprehensive molecular analysis on TNF superfamily genes reveals that a high TNF family signature predicts a worse prognosis, an immunosuppressive state, and a weak immunotherapy response [77]. Lowering tumor TNF cytotoxicity threshold can augment the immunotherapy impact [78]. Conversely, a recent study has reported that patients treated with immune checkpoint treatment who receive anti-TNF therapy actually fare worse [79]. Thus, the role of anti-TNF treatment in patients receiving immunotherapy requires further elucidation.

## Concluding comments

TNF has been intensively investigated for decades. Substantial progress has been made in understanding the structure of TNF and its receptors, and the signaling networks involved in TNF signaling. TNF is a key mediator of inflammation in rheumatologic and inflammatory diseases and anti-TNF treatments are highly effective. There is considerable information about the role of TNF in inducing cell death or cell survival, but often such information is derived from experimental models and needs validation in human tumors. A current important area of investigation is how TNF in tumors and TNF in the microenvironment of tumors influences the biology of cancers. TNF may also play an important role in mediating resistance to targeted inhibition, particularly in the context of EGFR inhibition in GBM and lung cancer. Whether TNF plays a role in resistance to targeting of other RTK nodes and/or in other tumor types is currently unknown. Future studies will elucidate these questions and also provide more insight into how anti-TNF therapy influences the response to immunotherapy.

## Methods

The recruitment criteria for TCGA data are as follows:

1. All primary NSCLC patients from TCGA-LUSC or TCGA-LUAD, the TCGA-LUAD EGFR-activating mutation was defined as harboring L858R or exon 19 deletion, but without T790M mutation. The recurrent NSCLC patients were excluded.
2. The patients must have RNAseq data.
3. The patients must have OS data.
4. The number of patients was displayed in Figure 1.

The recruitment criteria of BATTLE trial data is as follows:

1. Erlotinib treatment arm in the BATTLE trial, which is NCT00410059.
2. From NCT00410059, the selection criteria are the same as our previous study [17]. Pre–erlotinib-treatment gene expression data were available from 28 patients who went on to be treated with erlotinib. Three patients within this group had a very short progression-free survival (<0.5 months) and overall survival (<1 month) after starting erlotinib and were excluded from the analysis, since they presumably relapsed before erlotinib could exert its effect.
3. *N* = 25.

## Data Availability

The results published here are in part based upon data generated by the TCGA Research Network: https://www.cancer.gov/tcga.

## CRediT authorship contribution statement

**Ke Gong:** Conceptualization, Data curation, Formal analysis, Visualization, Writing - original draft. **Gao Guo:** Data curation, Formal analysis. **Nicole Beckley:** Writing - review & editing. **Yue Zhang:** Formal analysis. **Xiaoyao Yang:** Formal analysis, Visualization. **Mishu Sharma:** Writing - review & editing. **Amyn A. Habib:** Conceptualization, Writing - original draft, Funding acquisition, Supervision, Resources.
